# Possible Relevance of Receptor-Receptor Interactions between Viral- and Host-Coded Receptors for Viral-Induced Disease

**DOI:** 10.1100/tsw.2007.166

**Published:** 2007-07-02

**Authors:** Luigi F. Agnati, Giuseppina Leo, Susanna Genedani, Diego Guidolin, Nicola Andreoli, Kjell Fuxe

**Affiliations:** ^1^ Section of Physiology, Department of Biomedical Sciences, University of Modena and Reggio Emilia, Modena, Italy; ^2^ Section of Pharmacology, Department of Biomedical Sciences, University of Modena and Reggio Emilia, Modena, Italy; ^3^ Section of Anatomy, Department of Physiology and Human Anatomy, University of Padova, Padova, Italy; ^4^ Department of Neurosciences, Karolinska Institutet, Stockholm, Sweden; ^5^ IRCCS San Camillo, Venezia, Italy

**Keywords:** virus, G-protein coupled receptor, receptor-receptor interaction, receptor mosaic, vertical molecular networks, horizontal molecular networks

## Abstract

It has been demonstrated that some viruses, such as the cytomegalovirus, code for G-protein coupled receptors not only to elude the immune system, but also to redirect cellular signaling in the receptor networks of the host cells. In view of the existence of receptor-receptor interactions, the hypothesis is introduced that these viral-coded receptors not only operate as constitutively active monomers, but also can affect other receptor function by interacting with receptors of the host cell. Furthermore, it is suggested that viruses could also insert not single receptors (monomers), but clusters of receptors (receptor mosaics), altering the cell metabolism in a profound way. The prevention of viral receptor-induced changes in host receptor networks may give rise to novel antiviral drugs that counteract viral-induced disease.

## INTRODUCTION

The importance of the G-protein coupled receptors (GPCR) clearly results from the datum that about 3% of the human genome encodes these proteins. Their key roles in intercellular communication have made them the most studied targets for drug development. It is also established that more than 30 human diseases depend on mutations of GPCR and, as pointed out by Schőneberg[1], this number is expected to increase with the progress of our knowledge on the conformations and functions of these receptors.

The current investigations are mainly focused on the activating and inactivating mutations in GPCR genes as responsible for different human diseases, including cancers.

As to the pathological actions of viruses, several β-viruses have "pirated" genes that encode GPCR to elude the immune system and redirect cellular signaling receptor networks[2]. Herpesviruses are grouped into α, β, and γ subfamilies according to their biological properties and genomic organization. To date, only α-herpesviruses do not appear to encode GPCR. The best studied are β-herpesviruses, namely human herpesvirus-6 (HHV-6) and HHV-7, human cytomegalovirus (HCMV), and members of the γ-herpesviruses, such as HHV-8 (also known as Kaposi's sarcoma-associated herpesvirus). Recently, the γ-herpesvirus Epstein-Barr virus (EBV) was also found to encode GPCR.

In fact, the HCMV encodes at least four GPCR, of which US28 and UL33 display constitutive activity in transfected, and also in HCMV infected, cells[2]. In addition to being constitutively active, US28 is also activated by a broad spectrum of chemokines. A characteristic aspect of both US28 and UL33 is the promiscuous G-protein coupling, whereas chemokine receptors activate primarily Gi/o proteins[3].

Thus, herpesviruses have not only pirated GPCR, but have also mutated them into constitutively active and promiscuous GPCR. This ability enables the virus to alter host cell homeostasis in the absence of ligand, creating the capacity to trigger specific signaling pathways that lead to cell proliferation, survival, and migration, hence favoring the likelihood of virus survival. For example, by increasing host cell proliferation, the virus enhances the size of its reservoir pool and, by eluding the immune response, it enhances its likelihood of survival. The advantage of pirating chemokine receptors rather than other GPCR is understandable since chemokine receptor signaling pathways are involved in immune regulation in host cells and may enhance survival and/or proliferation. Through this pirating appropriation, the virus can redirect immune responses that otherwise could be potentially dangerous for its survival[3].

Let us summarize some of the main aspects of this subject to give a background of our proposal. The HCMV-encoded receptor US28 is not only active constitutively, but can also be activated further by several C-C chemokines, in particular by CX3C chemokine fractalkine (CX3CL1). Ligand binding to US28 causes continuous internalization of the receptor-ligand complex, hence sequestering chemokines. This process facilitates immune evasion at sites of infection and contributes to the latent presence of the virus. Signaling pathways that are activated in US28-expressing cells have been studied and it has been shown that, depending on the cellular-expression system, US28 elicits both constitutive and ligand-dependent signaling through multiple G proteins (see also above and Couty and Gershengorn[3]). Accordingly, several biological effects of US28 have been demonstrated. In particular, US28 induces migration of vascular smooth muscle cells, which might contribute to viral dissemination and the development of vascular disease. In atherosclerosis, it is tempting to speculate that US28 and UL33 cooperate to transform smooth muscle cells by activating mitogen activated protein kinases (MAPKs) and p38 MAPK- and p42/44 MAPK-dependent proliferative signaling pathways. Thus, cross-talk in signaling by UL33 and US28 during the course of HCMV infection occurs. As discussed below, the mechanism underlying their cross-talk may, at least in part, be receptor-receptor interactions. In any case, cellular proliferation and transformation caused by constitutive signaling of these viral GPCR enables the virus to increase the size of its reservoir pool and increases the number of cells that might potentially be infected. Moreover, acquisition of a transformed phenotype also induces infected cells to secrete soluble factors that, in turn, promote angiogenesis and inflammation, and inhibit the immune response.

The importance of these findings to better understand the pathologies induced by these viruses is clear, but these mechanisms can be discussed in a broader context, namely taking into account the receptor-receptor interaction mechanism (for a review see Agnati et al.[4]).

Thus, the present paper suggests new aspects on the possible role of viral GPCR on human diseases. Our proposal is based on the relevance of receptor-receptor interactions and of receptor mosaics or, more in general, of protein mosaics for GPCR decoding[5,6,7,8,9,10]. In particular, the main focus of the paper is on possible alterations in receptor mosaic (RM) composition and function induced by viral GPCR as a pathogenic mechanism.

## GPCR COULD FORM ABNORMAL RM

The RM has been defined as a cluster of receptors (R) working as an integrated input unit according to the types of receptors, the order of activation, and the topology of the assembly. The RM is connected to other proteins in the plane of the membrane, forming horizontal molecular networks (HMN), and to proteins reaching towards the extracellular or the intracellular environments, forming vertical molecular networks (VMN) (see Fig. 1[11,12]). Summing up, the integrative function of a RM is dependent on[13]:

1. Composition, i.e., by number and types of single R that form the mosaic
2. Spatial organization, i.e., the topology of the R inside the RM
3. Order of activation, i.e., the temporal sequence according to which the single R are activated
4. Interconnections with other molecules in the plane of the membrane (i.e., interconnections within the HMN) and in the extra- and/or intracellular environment (i.e., interconnections within the VMN)

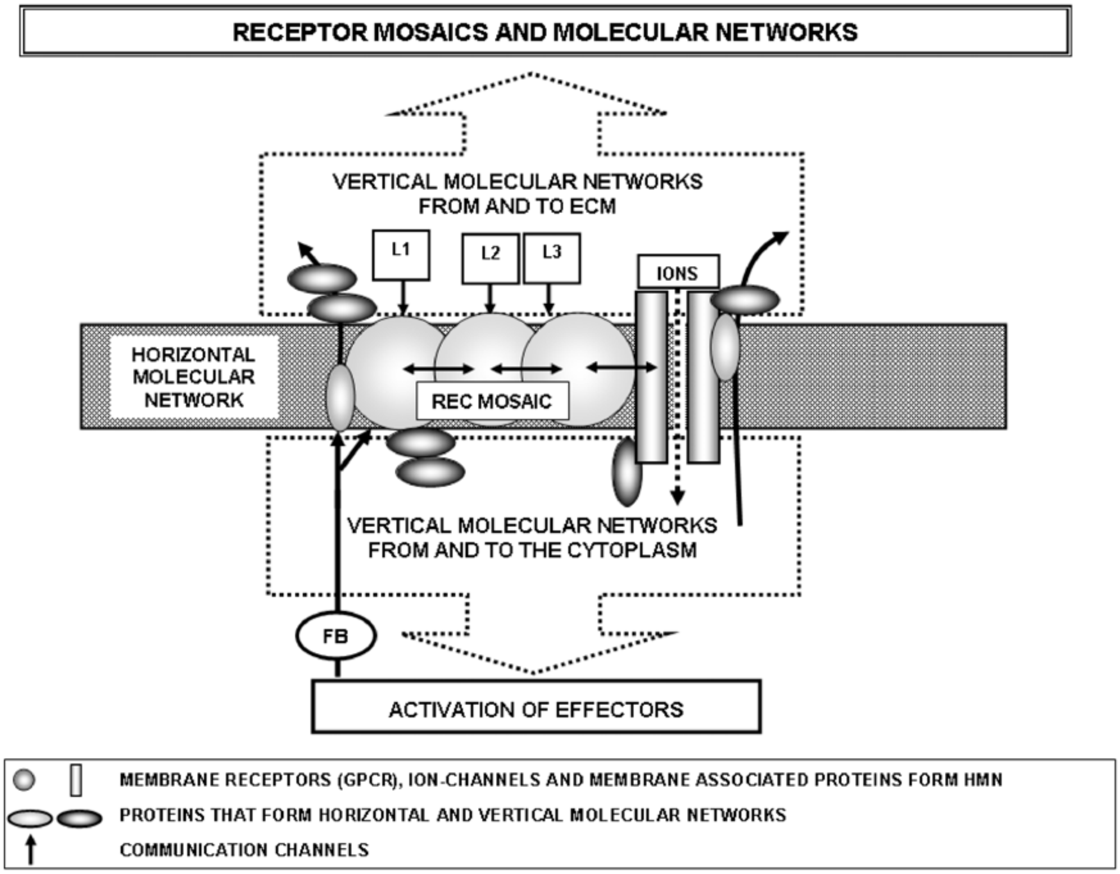

**FIGURE 1**. Schematic representation of the concept of horizontal and vertical molecular networks and of receptor mosaics. For further details, see text.

The order of activation of receptors within the cluster is probably highly relevant, but real data on this special aspect are missing at the moment. The relevance of composition can be deduced by the fact that some GPCR are not functionally competent if they are not interacting with the suitable partners. On the other hand, topology is also of the highest importance as it can be deduced by the special case of cooperativity among receptors in a RM[13,14,15,16].

In a relatively high number of cases, altered RM could have a pathological impact on cell function in view of the different aspects covered under 1–4 that affect the RM function.

## NORMAL GPCR COULD FORM ABNORMAL RM WITH VIRUS-CODED RECEPTORS

As pointed out in the Introduction, several β-viruses encode GPCR and can insert these receptor molecules into the host cell membrane on transfection. Some of these receptors seem to be constitutively active. It is possible to distinguish the approach and tethering of the virus to the host cell from the entry and integration with the genome of the cell. It has been shown that HCMV viron adhesion to the host cell occurs via CX3CL1-US28 high-affinity binding (see Fig. 2). The chemokine CX3CL1 (fractalkine, FK) is a novel multidomain protein expressed on the surface of cells. It has multiple functions and exists as two distinct forms: a membrane-anchored protein and a soluble chemotactic peptide that is cleaved from FK on the cell surface. As a full-length transmembrane protein, FK binds cells expressing CX3CR1, its cognate receptor, with high affinity. Proteolytic cleavage of FK releases a soluble form that is a potent chemoattractant for monocytes, T cells, and natural killer cells. Activation of protein kinase C dramatically increases the rate of this cleavage. Regulation of FK cleavage is critical for maintaining the balance between the immobilized and soluble forms[17]. The membrane form of CX3CL1, together with its only known receptor, CX3CR1, is highly expressed in neurons and microglial cells, and can be converted to the soluble form by the activation of two different metalloproteases[18]. As shown in Fig. 2, CX3CL1 has a crucial role for viral infection of the host. However, the possibility of a receptor-receptor (rec-rec) interaction, e.g., via the N-termini, favoring the tethering process should not be discarded. The present investigations are focused on the effect of these exogenous decoding molecules on the information handling by the cell. Our proposal considers also the possible role of receptor-receptor interactions in the viral-induced pathological mechanisms. Thus, it may be of relevance to investigate:

- The role of rec-rec interactions for the association of the viral GPCR to a cellular GPCR and the consequent alteration of normal cell receptors caused by rec-rec interactions with the viral GPCR (see Fig. 2).
- The association of the viral GPCR to a cellular RM and the consequent profound alteration of the complex decoding pathways controlled by that RM. As a matter of fact, it should be considered that the viral GPCR is usually constitutively active, thus the influences can be tonic and affect both intracellular as well extracellular VMN (see Fig. 3). The concept is further illustrated in Fig. 4, where the result of a numerical simulation of a RM behavior is shown.
- The insertion of an entire viral RM. Thus, it has not yet been investigated whether the β-virus–encoded GPCR are always present as monomers or oligomers, and if they are assembled as monomers or if they form RM in the infected host cell. The insertion of a viral RM is above all if connected with a suitable molecular effector, e.g., an ion-channel may lead to the formation of an abnormal HMN (RM interact with membrane proteins like G proteins ,scaffholding and adapter proteins, ion channels, and enzymes that together form the HMN in the lipid rafts of the surface membrane, see Agnati et al.[13]).

A possible experimental approach to the study of these aspects could be realized, for instance, by infecting a cell with a virus carrying a suitable GPCR, which in normal conditions is not present in the cell membrane. This would allow not only the detection of changes in the response of the receptor systems of the cell as a consequence of the insertion of the viral protein, but also the analysis (e.g., by means of FRET and/or colocalization software[19,20]) of the interaction at the membrane level between viral and cellular GPCR.

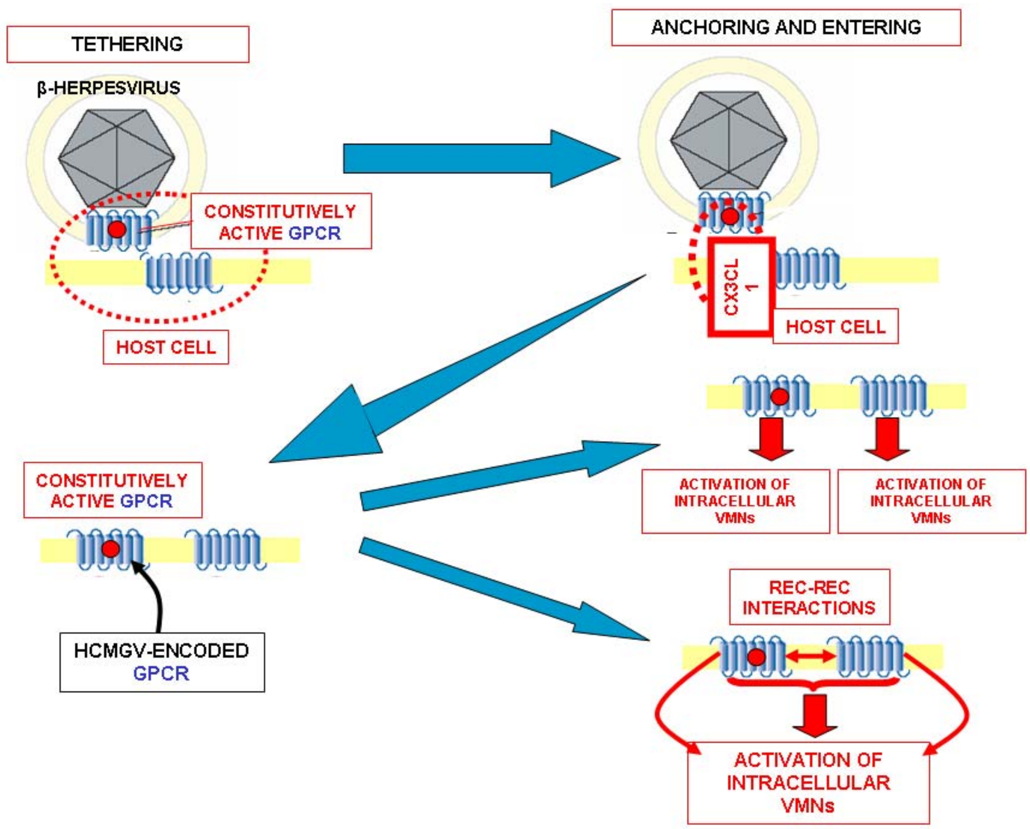

**FIGURE 2.** A virus-carried GPCR can cause pathology via rec-rec interactions with a host cell GPCR receptor. For further details, see text.

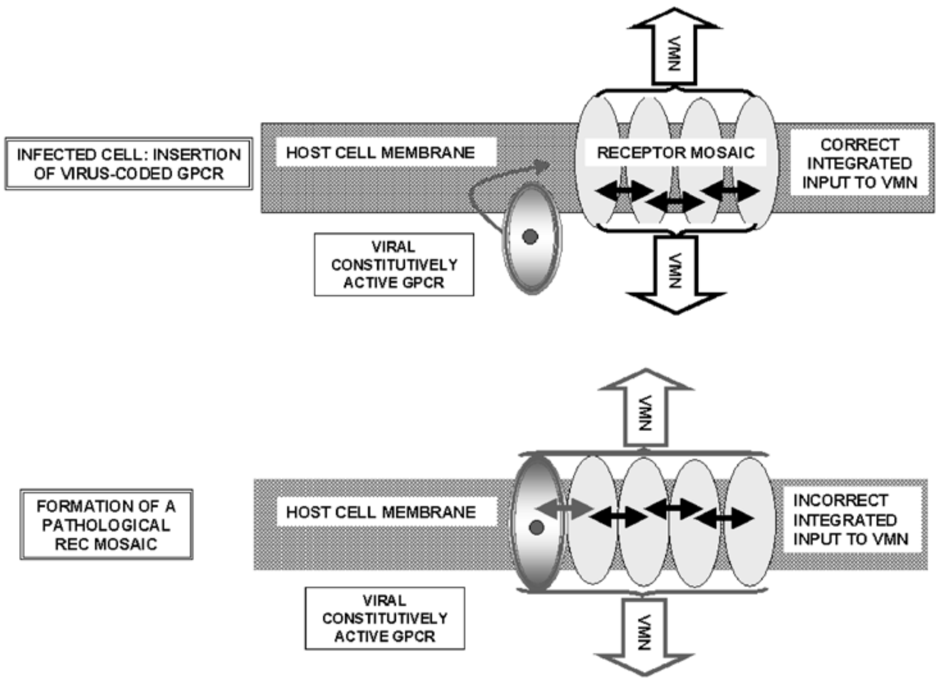

**FIGURE 3.** A virus-carried GPCR can be inserted into the host cell plasma membrane and redirect a host cell RM. For further details, see text.

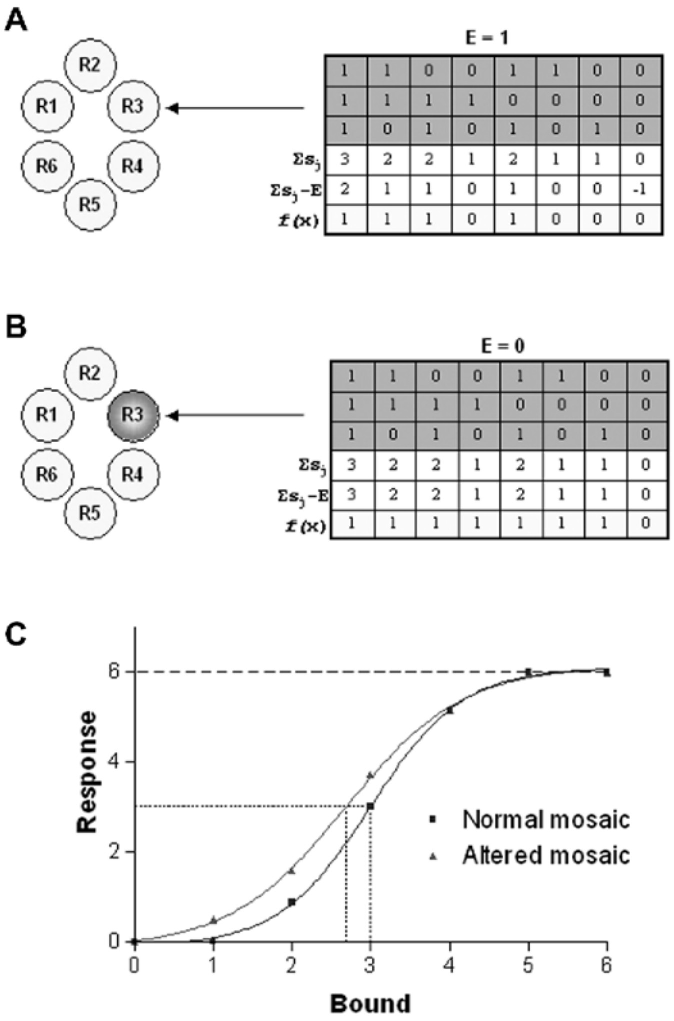

**FIGURE 4.** Mathematical modeling of a RM consisting of a ring of n = 6 receptors. Each receptor can assume inactive conformations (R states, all coded by 0 and characterized by a "low-affinity state" for the macromolecular effectors) and active conformations (R* states, all coded by 1 and characterized by a "high-affinity state" for the macromolecular effectors). The state($s(t)$) of each receptor evolves in the time as a function of its actual state and of the states of the two nearest neighbors with which it is in contact. A simple boolean function describing the change of receptor state as an effect of its interactions can be written as follows[24,25]:

$$s(t+1) = 1 \text{ if } \quad \sum s_j(t) > E$$
$$s(t+1) = 0 \text{ if } \quad \sum s_j(t) \le E$$

where E is threshold for receptor activation and the sum accounts for three terms (or inputs), the actual state of the receptor under scrutiny and the states of its two nearest neighbors. (A) Schematic view of the receptor cluster in its physiological arrangement, with six identical units. The assumed switching rule is illustrated on the right as a table where in the first row, the possible configurations of the three inputs are shown, while the output is shown in the last row. This switching rule (corresponding to E = 1) states that a receptor will become active if at least two of the three inputs it receives are in the active state. The same rule holds for all the receptors in the mosaic. (B) Schematic view of the receptor cluster with a single viral GPCR inserted in place of a normal receptor. The normal receptors will behave according to the rule illustrated in A. Conversely, the viral protein is assumed to be characterized by a lower activation threshold (E = 0). Thus, it becomes easier for it to be in the active state. This leads to the switching rule shown in the table on the right, stating that a single active input is sufficient to drive the viral protein into an activated state. (C) Response of the RM to a ligand. In the numerical simulation, the mosaic was initially set to an inactivated state. An increasing number ($n_b$) of receptors was then bound (i.e., switched to the state "1") and for each $n_b$ the mosaic response was recorded in terms of number of receptors ($n_a$) that are subsequently activated as an effect of the internal dynamics of the mosaic. Since there are many ways to choose $n_b$ receptors over n = 6 available units, all the possibilities corresponding to a given $n_b$ were explored and the obtained $n_a$'s averaged to provide the final estimate of the mosaic response. As illustrated, in both the analyzed conditions the result are sigmoid dose-response curves. However, the mosaic modified by the insertion of the viral protein exhibit a greater sensitivity to the incoming signal, as indicated by the ~15% decrease of the curve mid-point.

## DISCUSSION

The paper presents some possible deductions based on the experimental evidence that several human diseases depend on GPCR abnormalities and on the existence of RM (receptor oligomers) that operate as integrated input units in HMN. Thus, the hypotheses are introduced that pathologies can be caused not only by mutations and/or alterations in single GPCR, but also by:

- Abnormal decoding of a normal GPCR due, for example, to rec-rec interactions with a virus-encoded GPCR
- Abnormal decoding of a RM via altered composition and/or spatial organization (topology) of receptor in the RM due, for example, to the insertion in the RM of a virus-encoded GPCR
- Insertion of even an entire virus-carried RM into the host cell membrane

The relevance of rec-rec interactions at plasma membrane level is of paramount importance for coordination between signaling pathways, as already pointed out by Agnati et al.[21]. Thus, for viruses, it is strategically important not simply to redirect single receptor function, but rather to affect RM function.

In agreement with this view, it has recently been demonstrated that HCMV glycoproteins gB and gH independently bind EGFR and integrin αvβ3, respectively. Then αvβ3 translocates to lipid rafts where it physically interacts with EGFR to induce coordinated and mutually synergistic activation of their signaling pathways[22]. As noted by Wang et al.[22], this process represents the building of a complex molecular network[11], where there is an HMN at membrane level (in a special domain: the lipid raft) with a special input system (the RM at least formed by the heteromer αvβ3-EGFR) and an output system, a VMN involving the downstream molecules Src and the p85 subunit of PI3-K.

In view of these data, it could be of interest to investigate whether viruses can code also for molecular effectors (such as ion channels coupled to GPCR) and are able to associate these effectors to normal RM in the host cell membrane leading to the formation of an abnormal HMN and thereby causing not yet identified pathologies.

If some of these hypotheses are proven, it could be possible to develop new therapeutic strategies; for example, to devise drugs capable of irreversibly binding to the virus-carried pathogenetic molecules. Accordingly, the synthesis of the first nonpeptidergic inverse agonist has been reported for the viral-encoded chemokine GPCR US28. This compound is not only an important tool to investigate the significance of US28-mediated constitutive activity during viral infection[23], but also could indicate new approaches to treat β-herpesvirus infections.

A last aspect to discuss is the suggestion of the possible insertion of an entire viral RM into a host cell plasma membrane. This event can have profound effects on cell function especially if this RM contains constitutively active R and if it selects, via a specific tethering/binding process, a suitable effector system already present on the host cell membrane. As a matter of fact, this process could lead to a complex constitutively active pathological decoding mechanism inserted into the plasma membrane of the host cell and, hence, to a new direction for several intracellular and possibly extracellular VMN. This condition can be conceived of as a viral-caused pathologic cellular syndromic response favoring the survival of the virus and, thus, virus-induced disease. With the term “syndromic response” we have, in previous papers, indicated a set of finalistic interconnected elementary responses that produce a complex integrated cellular response as output[11].

## ACKNOWLEDGMENTS

This work was supported by grants from IRCCS Istituto di Neuroriabilitazione Motoria San Camillo, Venezia, Italy and from MIUR (PRIN 2004) Roma, Italy.
